# ZIF-67-Derived Flexible Sulfur Cathode with Improved Redox Kinetics for High-Performance Li-S Batteries

**DOI:** 10.3390/molecules29081833

**Published:** 2024-04-17

**Authors:** Chen Cheng, Hanyan Wu, Xinyang Chen, Shuiping Cai, Yingkang Tian, Xiaofei Yang, Xuejie Gao

**Affiliations:** 1 Center for Lignocellulosic Chemistry and Biomaterials, College of Light Industry and Chemical Engineering, Dalian Polytechnic University, Dalian 116034, China; cc15298509429@163.com (C.C.); wuhy1904@163.com (H.W.); cxysmail1@gmail.com (X.C.); caishuiping1086@163.com (S.C.); m15304113180@163.com (Y.T.); 2 Division of Energy Storage, Dalian National Laboratory for Clean Energy, Dalian Institute of Chemical Physics, Chinese Academy of Sciences, 457 Zhongshan Road, Dalian 116023, China; yangxf@dicp.ac.cn

**Keywords:** ZIF-67@CL cathode, electrochemical kinetics, catalyze the conversion of polysulfides, shuttle effect

## Abstract

Lithium–sulfur (Li-S) batteries have received much attention due to their high energy density and low price. In recent years, alleviating the volume expansion and suppressing the shuttle effect during the charge and discharge processes of Li-S batteries have been widely addressed. However, the slow conversion kinetics from polysulfide (LiPSs) to Li_2_S_2_/Li_2_S still limits the application of Li-S batteries. Therefore, we designed a ZIF-67 grown on cellulose (named ZIF-67@CL) as an electrocatalyst to improve the interconversion kinetics from LiPSs to Li_2_S_2_/Li_2_S for Li-S batteries. Based on the results of adsorption experiments of LiPSs, ZIF-67@CL and CL hosts were immersed in Li_2_S_4_ solution to adsorb LiPSs, and the UV-Vis test was conducted on the supernatant after adsorption. The results showed that the ZIF-67@CL had a stronger adsorption for LiPSs compared with the cellulose (CL). Furthermore, in the Li_2_S nucleation tests, the fabricated cells were galvanostatically discharged to 2.06 V at 0.112 mA and then potentiostatically discharged at 2.05 V. Based on the results of Li_2_S nucleation tests, the catalytic effect of ZIF-67 was further verified. As a result, the sulfur cathode used a ZIF-67 catalyst (named S/ZIF-67@CL) and delivered an initial capacity of 1346 mAh g^−1^ at a current density of 0.2 C. Even at a high current density of 2 C, it exhibited a high-capacity performance of 1087 mAh g^−1^ on the first cycle and maintained a capacity output of 462 mAh g^−1^ after 150 cycles, with a Coulombic efficiency of over 99.82%.

## 1. Introduction

Lithium–sulfur (Li-S) batteries, with high theoretical gravimetric energy density (2600 Wh kg^−1^) [1], low cost, and environmentally friendly nature, stand out among many rechargeable energy storage devices [2,3,4,5,6]. Nevertheless, several critical challenges hinder the application of Li-S batteries, including slow conversion kinetics, the shuttle effect, and huge volume expansion (80%) [7]. The existence of these problems reduces the utilization of active sulfur during the cycles, leading to irreversible capacity fading with low Coulombic efficiency. Over the past ten decades, most efforts have been devoted to solving the shuttle effect caused by polysulfides (LiPSs) and the volume expansion during the lithiation process to improve sulfur utilization and cell safety [8,9,10,11]. However, the sluggish conversion redox kinetics of LiPSs induced by the low conductivity of S/Li_2_S has found no effective solution, which is a primary reason for the rapid loss of sulfur species and capacity decay of Li-S batteries [12]. Therefore, accelerating interconversion kinetics between sulfur species is necessary to improve sulfur utilization.

Different from other catalytic redox reactions, such as oxygen reduction reaction (ORR) [13] and oxygen evolution reaction (OER) [14], the sulfur redox reaction (SRR) is complex. It undergoes a multi-phase transition from the solid phase (S_8_) to the liquid phase (LiPSs) and, finally, to the solid phase (Li_2_S_2_/Li_2_S) [15]. In the first phase, the potential values are above 2.1 V and S_8_ molecules on the cathode are reduced to long-chain LiPSs in this process, which provides 25% of the theoretical capacity (418 mAh g^−1^) for the Li-S battery with a fast kinetic [16]. Subsequently, the voltage is further reduced, and the liquid LiPSs are further reduced to solid Li_2_S_2_/Li_2_S at the second discharge platform. During this process, the Li-S battery contributes to another 75% of the theoretical capacity, which is equivalent to the specific capacity of 1256 mAh g^−1^ [17]. However, due to the high nucleation energy barrier of solid-state Li_2_S_2_ and the low conductivity of Li_2_S, the sulfur species conversion kinetics in this process is usually slow, reducing the utilization of sulfur. Therefore, the practical capacity of Li-S batteries is far from the theoretical value. Moreover, the shuttle effect produced by soluble LiPSs further aggravates the loss of active materials, as well as leads to corrosion of the positive active lithium [18,19]. Based on these considerations, accelerating the electrochemical conversion of LiPSs in the second discharge platform and alleviating the shuttle of soluble LiPSs are of paramount importance to improve sulfur utilization, as well as increase the reversible cycle capacity of the battery.

Recently, studies have demonstrated that the application of metal-organic frameworks (MOFs), such as ZIF-67, to Li-S batteries can effectively accelerate the LiPSs electrochemical conversion in the secondary discharge platform [20,21]. ZIF-67 is composed of organic ligands (2-Methylimidazole) with controllable metal centers (Co^2+^), which allows the open metal site Co^2+^ in ZIF-67 to exhibit Lewis acidic characteristics and catalyze the conversion of LiPSs to Li_2_S_2_/Li_2_S [22,23]. Studies have effectively catalyzed LiPSs to Li_2_S_2_/Li_2_S. These cannot effectively adsorb LiPSs, and they mitigate volume expansion during the charging and discharging processes, leading to a serious shuttle effect and volume expansion. To solve the above questions, recent studies have shown that cellulose (CL) contains a large number of hydroxyl groups in its structure, which can effectively adsorb LiPSs and thus inhibit the shuttle effect [24,25]. Moreover, cellulose has excellent flexibility that can mitigate the volume expansion during charging and discharging processes [26,27]. Therefore, combining ZIF-67 with flexible materials as cathodes for Li-S batteries provides a good solution to the problem of lithium–sulfur batteries [28,29].

Herein, we propose a novel approach to use ZIF-67 grown on cellulose as a host (hereafter referred to as ZIF-67@CL) as an effective catalyst for Li-S batteries. In this work, cellulose material was employed as a self-supporting carrier for Li-S batteries; both can physically adsorb LiPSs and alleviate the volume expansion during the charging and discharging processes [27]. Meanwhile, benefiting from the catalyzing of ZIF-67, the conversion kinetics of LiPSs to Li_2_S_2_/Li_2_S was propelled. Based on Li_2_S nucleation tests, the catalytic effect of ZIF-67 was demonstrated. In addition, the adsorption experiments of LiPSs effectively demonstrated the superior adsorption effect of cellulose carriers on LiPSs, while ZIF-67 had a strong adsorption effect on LiPSs. As a result, the Li-S batteries assembled with S/ZIF-67@CL electrodes delivered an initial high capacity of 1346 mAh g^−1^ at a current density of 0.2 C, which was significantly higher than that of S/cellulose (S/CL) electrodes (816 mAh g^−1^). Additionally, even at a high current density of 2 C, the S/ZIF-67@CL electrodes reached a high capacity of 1087 mAh g^−1^ on the first cycle and still retained 462 mAh g^−1^ cycled over 150 cycles, with a Coulombic efficiency of over 99.82%.

## 2. Results and Discussion

### 2.1. Schematic

**Figure 1:**
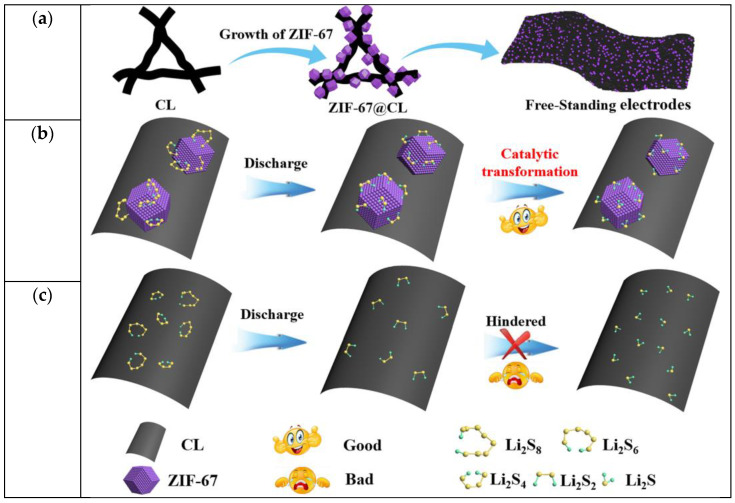
(**a**) Schematic illustrations of the synthesis process of ZIF-67@CL; schematic illustrations of sulfur species transformation on (**b**) ZIF-67@CL and (**c**) CL.

The fabrication of the ZIF-67@CL host is displayed in Figure 1a. Initially, cellulose served as a carrier material for ZIF-67 and was infiltrated in a solution containing cobalt nitrate to combine Co^2+^ with the hydroxyl groups. Afterward, a solution containing 2-methylimidazole was added to the above reaction to grow ZIF-67 on the surface of the cellulose (the loading of ZIF-67 was about 10 wt%). Acetylene black was added as the conductive additive in the reaction, and after vacuum filtration and drying, the free-standing hosts of ZIF-67@CL were obtained, serving as a flexible self-supporting sulfur host for Li-S batteries. The counterpart without ZIF-67 was denoted as CL. Further details regarding the synthesis process can be found in the experimental section. In this synthesized carrier material, cellulose provided the host with excellent free-standing properties due to its flexibility. Meanwhile, cellulose exhibited excellent electrolyte wettability, and the presence of hydroxyl groups further trapped LiPSs [24], inhibiting the shuttle effect. As depicted in Figure 1b,c, compared with CL, ZIF-67@CL effectively catalyzed the conversion from LiPSs to Li_2_S_2_/Li_2_S. This effect was attributed to the open metal site Co^2+^ in ZIF-67 exhibiting Lewis acidic characteristics, which further catalyzed the conversion of LiPSs [29]. Electrochemical studies further verified the positive effect of ZIF-67 in improving electrochemical performances.

### 2.2. Materials Characterization

**Figure 2:**
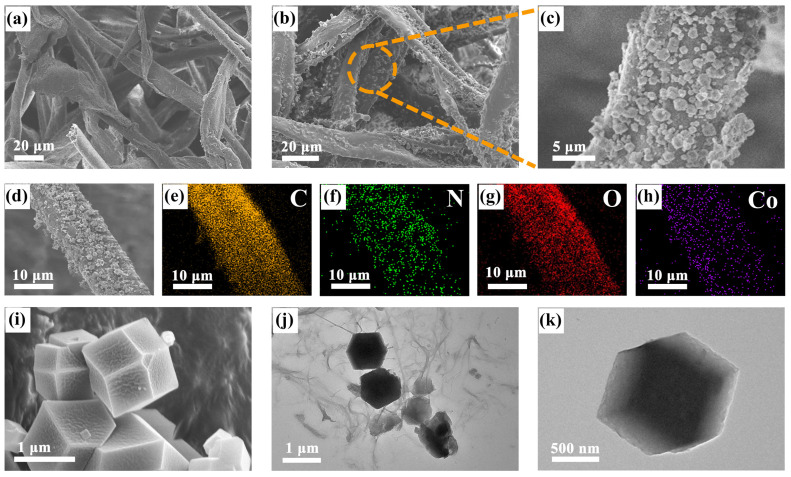
(**a**) SEM images of CL; (**b**–**h**) ZIF-67@CL at different magnifications and corresponding elemental mappings of C, N, O, and Co; (**i**) SEM images of ZIF-67 powder; TEM of (**j**) ZIF-67@CL and (**k**) ZIF-67 powder.

Before conducting electrochemical testing, material characterization was first carried out. First, the morphology of the products was studied using scanning electron microscopy (SEM). As presented in Figure 2a, the natural cellulose surfaces without any decoration were cross-linked with each other, showing a diameter of about 20 μm. This structure formed a three-dimensional electrical network and promoted deeper electrolyte penetration. Moreover, as shown in Figure S1a, CL enlarged into a single fiber gradually showed that the surface of natural cellulose was very smooth. By contrast, as displayed in Figure 2b,c, the modified ZIF-67@CL surface under different magnifications showed that the originally smooth fiber surface was loaded with many nano-boxes. These nano-boxes were about 1–1.5 μm in size, which were uniformly dispersed and covered the fiber surface. To verify that these nano-boxes were derived from ZIF-67, the pure ZIF-67 powder was observed via SEM. As presented in Figure 2i, the results demonstrated that the structure of ZIF-67 material consisted of nano-boxes and the sizes were approximately 1–1.5 μm, which showed the same structure as that covering the cellulose, identifying the successful growth of ZIF-67 on the cellulose. Moreover, the corresponding energy dispersive spectrometer (EDS) from the SEM further verified these results. As illustrated in Figure 2d–h and Figure S2, the EDS results of ZIF-67@CL and ZIF-67 showed the same presence of the elements C, N, O, and Co, and all of the elements were evenly distributed. However, due to the absence of ZIF-67 coverage, the CL had only two elements of C and O, respectively, as shown in Figure S1b,c. Additionally, transmission electron microscopy (TEM) measurements were also carried out to provide deep structural and compositional insights into the ZIF-67@CL. The TEM images of the ZIF-67@CL host and ZIF-67 powder are shown in Figure 2j,k, respectively. Compared with the TEM images of pure cellulose shown in Figure S3, the growth of ZIF-67 on cellulose was visible. ZIF-67, with a size of 1–1.5 μm, exhibited a dodecahedral structure, which was consistent with the SEM observations. Apparently, these ZIF-67 nano-boxes, which grew on the CL, acted as microreactors for trapping and conversion of LiPSs. In addition, the 3D CL served as a “bridge” connected each reactor and alleviated the volume expansion during the charging and discharging process.

### 2.3. Catalysis and Adsorption of LiPSs

**Figure 3:**
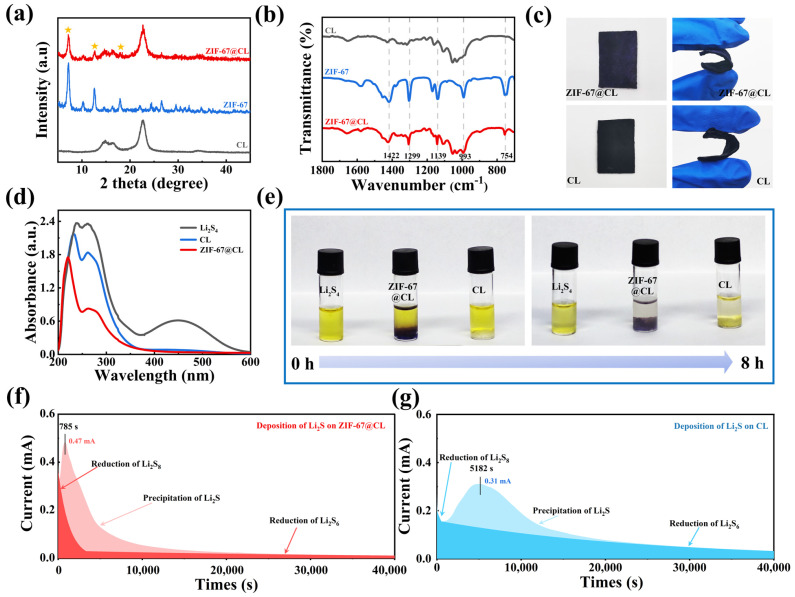
(**a**) Typical XRD patterns and (**b**) FT-IR results of the ZIF-67@CL, (**c**) ZIF-67 powder, and CL; (**d**) UV-Vis adsorption spectra of different simples immersed in Li_2_S_4_ for 8 h; (**e**) digital photographs of LiPSs adsorption experiment; Li_2_S nucleation tests for (**f**) ZIF-67@CL and (**g**) CL.

X-ray diffraction (XRD) and Fourier transform infrared spectrometer (FT-IR) tests were also used to explore the structure and components of the ZIF-67@CL composites. The XRD analysis confirmed the lattice structure of ZIF-67 on the CL. Specifically, as shown in Figure 3a, the XRD patterns of ZIF-67@CL showed intense peaks at 7.2°, 12.68°, and 18.0° (Denoted by star symbols in the Figure 3a), which were in agreement with the diffraction peaks of pure ZIF-67 (simulated) [30,31,32]. This result indicated the presence of crystallized ZIF-67 in ZIF-67@CL composites. Furthermore, the broad peaks of 14–16° and the characteristic peak of 22.5° of CL matched the characteristic peaks of natural cellulose [33]. Afterward, the FT-IR test further demonstrated the internal chemical structures of ZIF-67@CL and CL.

The FT-IR absorption peaks of ZIF-67@CL were comparable to pure CL and are illustrated in Figure 3b. Based on the results of FT-IR, the multiple prominent absorption peaks at 754, 993, 1139, 1299, and 1422 cm^−1^ were attributed to the bending and stretching vibration peaks of the ZIF-67 crystal [34]. These findings agreed with the XRD results and illustrated the successful loading of the ZIF-67 on the CL. Finally, flexibility experiments were conducted to verify the ability of the synthesized materials ZIF-67@CL and CL to reduce volume expansion. As shown in Figure 3c, the synthesized materials (ZIF-67@CL and CL) were bent nearly 180 degrees, demonstrating their excellent flexibility and self-supporting properties. Compared with the rapid capacity attenuation caused by volume changes during battery charging and discharging that occurred on coated current collectors, the unique flexibility of cellulose could reduce the degree of volume changes, thereby improving the stability of the battery during cycling.

In typical Li-S batteries, the shuttle of soluble LiPSs leading to low sulfur utilization was one of the main reasons for the rapid capacity fading. Improving the adsorption of LiPSs in the cathode material was conducive to suppressing the shuttle effect in Li-S batteries, which effectively enhanced the performance of the Li-S batteries. Therefore, the LiPSs static adsorption experiments of ZIF-67@CL and CL were conducted to explore the effects of both hosts in terms of inhibiting the shuttle effect. In this work, a 0.03 M Li_2_S_4_ solution was used for the soluble LiPSs, and ultraviolet and visible (UV-Vis) spectra were used to analyzed the LiPSs content in the solution after adsorption. As shown in Figure 3e, ZIF-67@CL and CL hosts were immersed into 1 mL Li_2_S_4_ solution, respectively, to adsorb LiPSs in the solution. After 8 h of adsorption, the solutions adsorbed by CL and ZIF-67@CL showed varying degrees of fading, which indicated the effective adsorption capacity of CL and ZIF-67@CL for LiPSs. The adsorption capacity of CL for LiPSs came from the presence of hydroxyl groups on cellulose. Polar hydroxyl groups could effectively adsorb LiPSs through chemical adsorption, thereby providing CL with the effective ability to suppress the shuttle effect [35]. However, it was clearly observed that the solution adsorbed by ZIF-67@CL showed a more obvious fading effect than CL, suggesting a stronger adsorption capability of ZIF-67@CL toward LiPSs. The UV-Vis test also verified the same result; as shown in Figure 3d, the LiPSs ions caused the pure Li_2_S_4_ solution (the black line) to present an adsorption peak around 250 nm [36,37], and the height of the peak was compared to the content of LiPSs ions. Notably, after the adsorption, the content of LiPSs in the solution decreased (the decrease of peak height), suggesting a reduction in the LiPSs content after adsorption. Moreover, the ZIF-67@CL host that adsorbed the Li_2_S_4_ solution still had the lowest peak intensity of LiPSs ions, implying that minimal LiPSs remained in the solution. This result indicated the stronger adsorption ability of ZIF-67@CL for LiPSs than CL. This was mainly due to the presence of the unique structure of nano-boxes of ZIF-67 on the ZIF-67@CL, which further enhanced the adsorption ability of ZIF-67@CL for LiPSs [38]. Based on the LiPSs adsorption experiments, it was proven that the synthesized composite carrier of ZIF-67@CL played an effective role in improving the LiPSs adsorption capacity of the sulfur host. This phenomenon was crucial to enhancing sulfur utilization in the Li-S batteries. Subsequently, Li_2_S deposition studies were carried out to investigate the impacts of ZIF-67 in enhancing the conversion of LiPSs. As shown in Figure 3f,g, 50 μL of cathodic electrolyte (0.2 M Li_2_S_8_ solution) was added to the positive side of ZIF-67@CL and CL, and 50 μL of Li-S electrolyte was added to the negative side. After discharging at 2.05 V constant potential, the time-dependent reduction current followed characteristic curves corresponding to Li_2_S deposition on different substrates, and the electric current was plotted in integral form. According to the LaMer theory [39], the “incubation” period began with a monotonically declining current caused by the electroreduction of long-chain LiPSs to Li_2_S_4_. The production of Li_2_S nuclei at the electrode interface was responsible for the following rise in current and the appearance of a current peak [40]. Notably, the current peak of the ZIF-67@CL was 0.47 mA, which was higher than the CL (0.31 mA), indicating a greater precipitation quantity of Li_2_S on ZIF-67@CL. Moreover, the response time of the ZIF-67@CL was also shorter (785 s of ZIF-67@CL, and 5182 s of CL). This demonstrated that ZIF-67 significantly improved redox reaction kinetics and responded to Li_2_S faster within a shorter period of time, which was mainly attributed to the ZIF-67 presenting a hollow nanocage structure that could effectively adsorb LiPSs. Also, the open metal site Co^2+^ in ZIF-67 demonstrated Lewis acidic properties and further catalyzed the conversion of LiPSs [37]. The Li_2_S nucleation results verified that the introduction of ZIF-67 on the CL enhanced the electrocatalytic activity of the host for LiPSs, which accelerated the deposition of Li_2_S and improved the capacity output in the Li-S batteries. As a result, the adsorption experiments and nucleation experiments proved the role of ZIF-67@CL in inhibiting the shuttle effect of soluble LiPSs and catalytically improving the conversion of sulfur species. These features effectively promoted sulfur utilization and elevated the cycling stability of the Li-S batteries.

### 2.4. Li Full Cells with Different Cathode Materials

**Figure 4:**
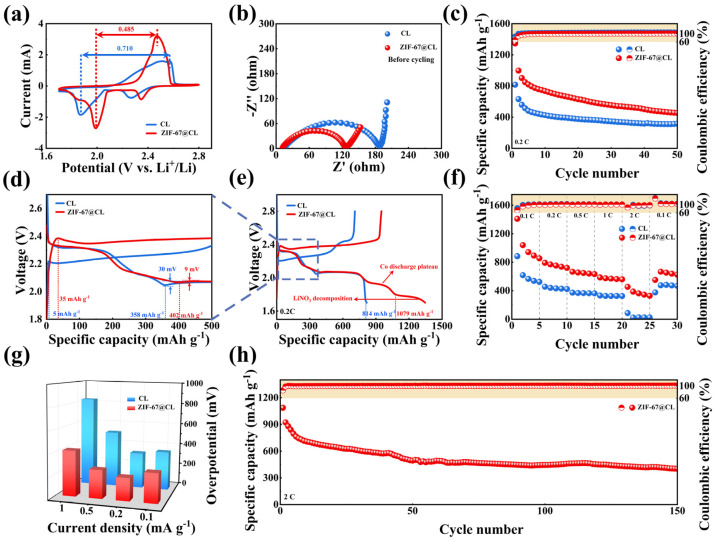
Electrochemical performances of the S/ZIF-67@CL and S/CL electrodes with a sulfur loading of 1.8 mg cm^−2^; (**a**) CV profiles; (**b**) EIS plots before cycling; (**c**) cycling performance at 0.2 C; (**d**) (**e**) charge/discharge curves at 0.2 C; (**f**) rate performance at various current densities ranging from 0.1 C to 2 C; (**g**) evolution of the overpotential at various current densities from 0.1 C to 2 C; (**h**) cycling performance at 2 C.

To verify the effect of ZIF-67 on improving the performance of Li-S batteries, the sulfur carriers employed the ZIF-67@CL and CL, respectively, to assemble the Li-S full batteries. The electrodes were named S/ZIF-67@CL and S/CL, with sulfur loading of 1.8 mg cm^−2^. Firstly, cyclic voltammetry (CV) curves were performed with sweep rates of 10^−4^ V s^−1^ within a potential window of 1.7–2.8 V. As shown in Figure 4a, the S/ZIF-67@CL and S/CL electrodes exhibited typical CV curves, with two cathodic and one anodic peak. In the CV cures, two peaks corresponded to the reduction of S_8_ molecules to long-chain LiPSs (2.34 V) and the further reduction of LiPSs to short-chain solid Li_2_S_2_/Li_2_S (1.98 V), respectively. Additionally, an oxidation peak at the anode side (2.46 V) represented a reversible oxidation process of Li_2_S_2_/Li_2_S to sulfur [41], while in the S/ZIF-67@CL, the additional plateau around 1.88 V was attributed to the reduction reaction of Co in ZIF-67 [42]. Also, as presented in Figure S4, the first cycle of the CV curve observed this phenomenon. Additionally, a peak around 1.7 V in the first CV curve of S/ZIF-67@CL corresponded to the decomposition of LiNO_3_ [41]. Compared to the CV curves, as presented in Figure 4a, the cathodic and anodic peaks of S/ZIF-67@CL were sharper than those of S/CL. It was suggested that the addition of ZIF-67 catalysts brought more electroactive sites to improve the sulfur species conversion. Moreover, the S/ZIF-67@CL cell had a larger integration area and smaller peak spacing than the S/CL cell (0.485 V of S/ZIF-67@CL and 0.710 V of S/CL), suggesting that the S/ZIF-67@CL electrode reduced the polarization and increased the specific capacity of Li-S batteries [42]. This phenomenon of reducing polarization in the battery was attributed to the rapid conversion behavior in Li_2_S nucleation, which was verified by the Li_2_S nucleation experiment. The improved reactivity was further supported by electrochemical impedance spectroscopy (EIS) plots. As shown in Figure 4b, before cycling the batteries, the S/ZIF-67@CL electrode exhibited smaller values of ohmic resistance (R_s_) and charge transfer resistance (R_ct_). The R_s_ values were 11.98 Ω and 16.08 Ω for S/ZIF-67@CL and S/CL, respectively. The R_ct_ values were 125 Ω and 186 Ω for S/ZIF-67@CL and S/CL, respectively. Additionally, as demonstrated in Figure S5, S/ZIF-67@CL also showed smaller R_s_ and R_ct_ values compared to S/CL after cycling, further confirming its faster electrochemical kinetics, thereby accelerating lithium-ion transfer rates and sulfur conversion. Therefore, the chemical reaction kinetics were enhanced by introducing the ZIF-67, which catalyzed electrochemical processes and improved the electrochemical activity during the charging and discharging processes.

The cycling stability of Li-S batteries was explored to highlight the advantages of ZIF-67 in catalyzing sulfur interconversion. As illustrated in Figure 4c, the cells with S/ZIF-67@CL electrodes exhibited a high initial capacity of 1346 mAh g^−1^ at 0.2 C, which was much higher than the S/CL electrodes, with an initial capacity of only 816 mAh g^−1^. This phenomenon demonstrated a high utilization of sulfur in the S/ZIF-67@CL electrodes. Furthermore, as shown in Figure S8, we conducted SEM analysis of the ZIF-67 material from the Li-S batteries after cycling. We found that ZIF-67 maintained its original nano-box-like structure even after the battery cycling. This result confirmed the excellent structural stability of ZIF-67, thus enhancing the cycle stability of the Li-S batteries. Furthermore, the chemical catalyst and physisorption of LiPSs by ZIF-67 and CL were in favor of the cycling stability of the S/ZIF-67@CL cathodes. Afterward, the curves, at a rate of 0.2 C, were provided to further explain the above conclusions. As shown in Figure 4e, the S/ZIF-67@CL electrode exhibited three plateaus in the voltage window of 1.7~2.8 V, consistent with the peaks of the CV curves. Specifically, two typical plateaus near 2.3 V and 2.1 V were attributed to the two-step reduction reaction from sulfur reduction to Li_2_S, and another plateau around 1.7 V corresponded to the decomposition of LiNO_3_. The additional plateau near 1.88 V was associated with the reduction reaction of Co in ZIF-67. Compared to S/CL electrode, excluding the capacity contribution of Co, the capacity of the S/ZIF-67@CL electrode was also as high as 1079 mAh g^−1^, which was much higher than the S/CL electrode (814 mAh g^−1^). This result demonstrated that ZIF-67 improved the LiPSs conversion, resulting in a higher sulfur utilization. This improvement could be attributed to the exposed, open metal Co^2+^ site from the ZIF-67 exhibiting Lewis acidic characteristics and catalyzing the interconversion of sulfur species [22]. Moreover, as displayed in Figure 4d, the detailed charge/discharge curves were presented at a rate of 0.2 C. From this result, the S/ZIF-67@CL electrode provided a capacity of 402 mAh g^−1^ at the first plateau around 2.3 V, showing an increase of 44 mAh g^−1^ compared to the S/CL electrode. This increase was mainly attributed to the interaction between ZIF-67 and LiPSs, which improved the content of LiPSs reduction. Furthermore, following the first discharge plateau, a distinct valley formed between the first and second plateaus, which was known as the Li_2_S nucleation point. The Li_2_S nucleation point was determined based on the potential difference between the tangential line of the potential plateau and the kinetics of Li_2_S nucleation [43]. Notably, the nucleation overpotentials of the S/CL electrode and the S/ZIF-67@CL electrode were 30 mV and 9 mV, respectively. The lower overpotential of the S/ZIF-67@CL electrode compared to the S/CL electrode suggested a lower interfacial energy barrier for Li_2_S nucleation and deposition on the surface of the S/ZIF-67@CL electrode. In other words, the nucleation behavior of Li_2_S was altered by the S/ZIF-67@CL electrode due to the loading of ZIF-67. This result indicated that ZIF-67 facilitated the nucleation and deposition process of Li_2_S [44], which was consistent with the conclusions of the Li_2_S nucleation experiments.

In addition, the C-rate performance of S/ZIF-67@CL and S/CL electrodes was measured at different current densities ranging from 0.1 to 2 C, as shown in Figure 4f. Figure S6a,b displays the corresponding charge/discharge curves. The results showed that S/ZIF-67@CL was able to maintain the typical charge/discharge plateau well, even at a high rate of 2 C, implying the ability of S/ZIF-67@CL to achieve fast-charging Li-S batteries. Moreover, the S/ZIF-67@CL electrode delivered almost double the discharge specific capacity than the S/CL electrode, and the average discharge specific capacities of S/ZIF-67@CL at 1 and 2 C were 570.84 and 375.44 mAh g^−1^, respectively. However, the average discharge specific capacities of S/CL were only 327.44 and 37.2 mAh g^−1^, respectively. Moreover, when the rate returned to 0.1 C, the capacity of the S/ZIF-67@CL reached 627.46 mAh g^−1^, indicating an excellent reversibility of the S/ZIF-67@CL. The improved performance of S/ZIF-67@CL was mainly due to the catalytic ability of ZIF-67, which effectively reduced the internal polarization of the battery. Furthermore, the charge/discharge curves of S/ZIF-67@CL and S/CL electrodes under different rates were investigated to further analyze the changes in the overpotential of the batteries. As illustrated in Figure 4g, the overpotential was determined by the voltage difference based on the median of the second discharge plateau at different current densities. The overpotential could simulate the electrochemical kinetics of sulfur species on various substrates [45]. Based on the calculation results of overpotential, the values of S/ZIF-67@CL electrode were 269.5, 208.3, 258.4, and 415.3 mV at 0.1, 0.2, 0.5, and 1 C, respectively. In comparison, the overpotential values of S/CL electrode were 358, 332.3, 520, and 834 mV at 0.1, 0.2, 0.5, and 1 C, respectively. The overpotential at a 2 C current density was not shown due to the short-circuit phenomenon of S/CL electrode at 2 C, which was unable to cycle at such a high rate, as shown in Figure S6b. As displayed in Figure 4g, the S/ZIF-67@CL electrodes exhibited smaller overpotential (almost about a half of S/CL electrodes) than the S/CL electrodes at all rates, suggesting the faster electrochemical kinetics of sulfur species and a reduction polarization phenomenon on the S/ZIF-67@CL electrodes. Finally, the long-life cycling stability of S/ZIF-67@CL electrodes at a high rate of 2 C was studied to verify the ability of S/ZIF-67@CL to quickly charge Li-S batteries. As shown in Figure 4h, the battery delivered a high capacity of 1087 mAh g^−1^ in the initial cycle and maintained a capacity of 462 mAh g^−1^ after 150 cycles, with a Columbic efficiency of over 99.82%. This result highlights the benefits of ZIF-67 in increasing sulfur usage and improving cycling stability by accelerating electrochemical kinetics and chemical interaction between LiPSs and ZIF-67.

## 3. Experimental

### 3.1. Materials

The experimental materials Co(NO_3_)_2_·6H_2_O (99.99%), 2-methylimidazole (98%), carbon disulfide (CS_2_) (99%), ethylene glycol dimethyl ether (DME) (99.5%) were purchased from Shanghai Aladdin Biochemical Technology Co., Ltd. (Shanghai, China). Acetylene black (99.9%) was purchased from Guangdong Canrd New Energy Technology Co., Ltd. (Dongguan, China). Methanol (99.5%) was purchased from Shanghai Macklin Biochemical Technology Co., Ltd. (Shanghai, China). The liquid electrolyte used in this study was 1.0 M LiTFSI in DOL:DME = 1:1 Vol% with 1.0% LiNO_3_, supplied by Guangdong Canrd New Energy Technology Co., Ltd. China (Dongguan, China). All chemicals were directly employed without further treatment.

### 3.2. Fabrication of ZIF-67@CL and CL Composite

Solution A was synthesized by dissolving 0.825 g Co(NO_3_)_2_·6H_2_O in 20 mL of methanol with stirring, and then placing 0.15 g cotton cellulose into it under magnetic stirring to afford a uniform dispersion for 12 h. Solution B was formed by dissolving 0.462 g 2-methylimidazole in 20 mL methanol. Then, solution B was added to solution A, and the mixture was stirred for 24 h at room temperature to form a purple solution. After 24 h of growth at room temperature, the resultant purple fiber was taken out of the ligand solution and washed with cold ethanol to remove the excess 2-methylimidazole with the help of a vacuum filtration device. The fibers with 20 mL ethanol were redistributed, and 0.15 g acetylene black was added to it and stirred for 48 h. Afterward, a vacuum filtration device was utilized to filter the fibers, and they were dried at 150 °C for 10 h. The thickness was pressed to 45 mm with a roller press. The harvested ZIF-67 which grew on the fibers was denoted as ZIF-67@CL. Similarly, the cotton cellulose exclusive of ZIF-67, denoted as CL, was also prepared by the same method and used for comparison.

### 3.3. Fabrication of S/ZIF-67@CL and S/CL Composite

Firstly, 20 mg sulfur was dissolved into 1 mL CS_2_ solution. The ZIF-67@CL and CL were cut into disks with diameters of 10 mm. The S/ZIF-67@CL and S/CL were obtained by directly absorbing 20 mg mL^−1^ CS_2_ with sulfur dissolved, and then the solvent was naturally removed at 70 °C 10 h in a vacuum oven. The different sulfur-loading electrodes were obtained by controlling the solution volume. The loading of the sulfur cathodes was 1.8 mg cm^−2^.

### 3.4. Materials Characterization

The morphology and structure of the cellulose and ZIF-67 were characterized via a scanning electron microscope (SEM) (Hitachi S–4800, Hitachi, Tokyo, Japan) equipped with an energy disperse spectrometer (EDS). Transmission electron microscopy (TEM) (JEM–2100, Tokyo, Japan) observed the surface morphology and characteristics further. The ZIF-67@CL and ZIF-67 powder and CL were investigated at a scan rate of 8 min^−1^ from 5° to 80° by X-ray diffraction (SHIMADZU XRD–6100, SHIMADZU, Tokyo, Japan). The Fourier transform infrared (FT-IR) spectra were measured in the range of 450–4000 cm^−1^, with a resolution of 4 cm^−1^, using a FT-IR Spectrometer (Spectrum Two, Waltham, MA, USA).

### 3.5. Electrochemical Measurements

The electrochemical performances of the S/CL and S/ZIF-67@CL electrodes were tested with CR2032 coin cells, constructed in an Ar-filled glove box. The S/CL and S/ZIF-67@CL electrodes were directly used as free-standing electrodes to assemble batteries. The cathode and Li anode were separated using a Celgard 2400 microporous polypropylene (PP) separator (Celgard, Charlotte, NC, USA). The electrolyte used in this study was 1.0 M LiTFSI in DOL:DME = 1:1 Vol% with 1.0%LiNO_3_. The electrolyte amount was controlled at 100 μL in each cell. The galvanostatic charge/discharge test and rate performance were tested using a battery test system (LAND CT3002A, Blue Electric Electronics Co., Ltd., Wuhan, China) within a voltage range from 1.7 to 2.8 V. Cyclic voltammetry curves and electrochemical impedance spectroscopy were measured by electrochemical workstations (CHI660E, Chenhua Instrument Co., Ltd., Shanghai, China). CV curves were performed with sweep rates from 10^−4^ V s^−1^. EIS profiles were tested with a frequency range from 10^−1^ to 10^5^ Hz.

### 3.6. Visualized Adsorption Test

For lithium LiPSs solution (mainly Li_2_S_4_, 0.03 M), the Li_2_S_4_ solution was prepared by mixing Li_2_S and sulfur with a molar ratio of 1:3 in DME, followed by vigorous magnetic stirring. Li_2_S_4_/DME test solution (7 mL) was obtained by mixing 6.0 mL of DME solvent and 1 mL of 0.03 M Li_2_S_4_ solution. ZIF-67@CL and CL host materials (600 mg) were immersed in 1 mL of Li_2_S_4_/DME solutions. All operations were conducted in a glovebox. All supernatants after treatment with various sulfur hosts were analyzed by UV-Vis spectroscopy.

### 3.7. Li_2_S Nucleation Test

A nucleation test of Li_2_S was conducted in CR2032 coin cells; 0.2 M Li_2_S_8_ was prepared by adding Li_2_S and S with a molar ratio of 1:7 into the DME solvent. ZIF-67@CL and CL were applied as the work electrode, Li foil worked as the counter electrode, 50 μL Li_2_S_8_ electrolyte was added in the positive side, and 50 μL of 1.0 M LiTFSI in DOL:DME = 1:1 Vol% with 1.0%LiNO_3_ solution was used as an anolyte. The cells were first discharged at a current of 0.112 mA to 2.06 V, and then the voltage was held at 2.05 V for a period of time for Li_2_S nucleation and growth [40,46].

## 4. Conclusions

In summary, we constructed a self-supporting carry with high sulfur catalytic properties (ZIF-67@CL) as a sulfur support for Li-S batteries through the double-solution impregnation method. Cellulose, as the supporting carrier, could adsorb LiPSs by means of hydrogen bonds, and the excellent flexibility of cellulose also mitigated the volume expansion during the charge/discharge processes. Additionally, according to the LiPSs adsorption results, the adsorption effect of LiPSs by the carrier was further improved after loading ZIF-67 nano-boxes on the cellulose. Meanwhile, the Li_2_S nucleation experiment demonstrated the high catalytic properties of ZIF-67 for the LiPSs, which enhanced the sulfur conversion and derived a high-capacity output of the S/ZIF-67@CL electrodes. As a result, the Li-S batteries of S/ZIF-67@CL electrode delivered a high initial discharge capacity of 1346 mAh g^−1^ at a current density of 0.2 C, which was significantly higher than the S/CL electrode (816 mAh g^−1^). Furthermore, even at a high current density of 2 C, the ZIF-67@CL electrodes exhibited a high capacity of 1087 mAh g^−1^ on the first cycle and maintained a capacity utilization of 462 mAh g^−1^ after 150 cycles. This work illustrates the critical role of ZIF-67 in enhancing the kinetics of electrochemical reactions and improving sulfur utilization in self-supporting Li-S batteries.

## Data Availability

The data presented in this study are available in the Supplementary Materials.

## Supplementary Materials

The following supporting information can be downloaded at: https://www.mdpi.com/article/10.3390/molecules29081833/s1, Figure S1: SEM images of (a) CL and (b,c) corresponding elemental mappings of C, O; Figure S2: SEM images of (a) ZIF-67 powder and (b–e) corresponding elemental mappings of C, N, O, Co; Figure S3: TEM images of CL; Figure S4: CV profiles at first cycle; Figure S5: EIS plots after cycling; Figure S6: Rate performance corresponding to charge/discharge profiles of (a) S/ZIF-67@CL and (b) S/CL; Figure S7: (a) S/ZIF-67@CL and (b) S/CL charge/discharge curves of different cycles at 0.2 C; Figure S8: SEM images of (a,b) ZIF-67@CL after Li-S battery cycling and (c–g) corresponding elemental mappings of C, N, O, Co, S; Figure S9: S/ZIF-67@CL charge/discharge curves of different cycles at 2 C.
