# Supplementary material for: ZIF-67-Derived Flexible Sulfur Cathode with Improved Redox Kinetics for High-Performance Li-S Batteries
Source: Molecules. 2024 Apr 17;29(8):1833. doi: 10.3390/molecules29081833 (PMC11052357; doi:10.3390/molecules29081833)
Supplement: Supplementary file 1 [file molecules-29-01833-s001.zip › molecules-2960038-supplementary.pdf]

Supporting Information

ZIF-67-Derived Flexible Sulfur Cathode with Improved Redox Kinetics for High-Performance Li-S Batteries

Chen Cheng [1], Hanyan Wu [1], Xinyang Chen [1], Shuiping Cai [1], Yingkang Tian [1], Xiaofei Yang [2] and Xuejie Gao [1,*]

1 Center for Lignocellulosic Chemistry and Biomaterials, College of Light Industry and Chemical Engineering, Dalian Polytechnic University, Dalian 116034, China; cc15298509429@163.com (C.C.); wuhy1904@163.com (H.W.); cxysmail1@gmail.com (X.C.); caishuiping1086@163.com (S.C.); m15304113180@163.com (Y.T.)
2 Division of Energy Storage, Dalian National Laboratory for Clean Energy, Dalian Institute of Chemical Phys-ics, Chinese Academy of Sciences, 457 Zhongshan Road, Dalian 116023, China; yangxf@dicp.ac.cn
* Correspondence: gaoxuejie1107@163.com

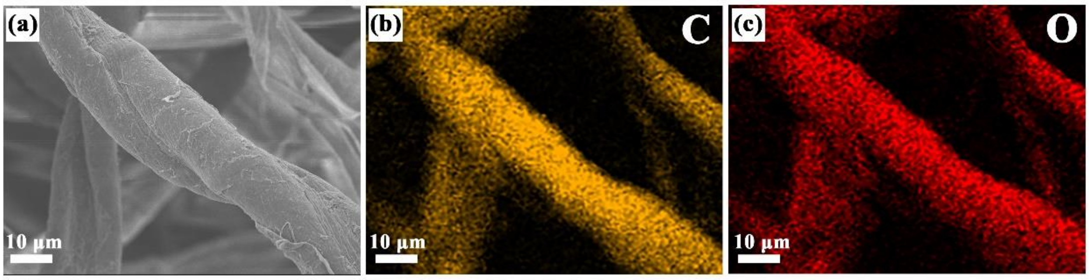

Figure S1: SEM images of (a) CL and (b) (c) corresponding elemental mappings of C, O.

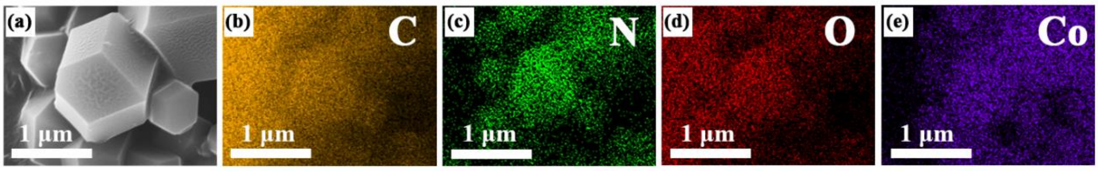

Figure S2: SEM images of (a) ZIF-67 powder and (b-e) corresponding elemental mappings of C, N, O, Co.

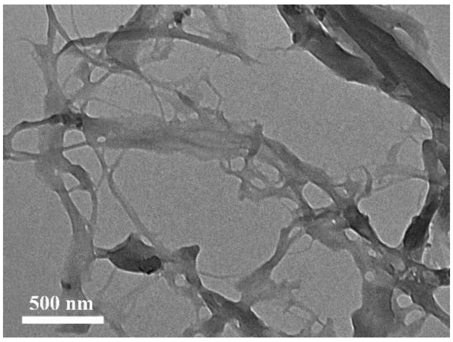

Figure S3: TEM images of CL.

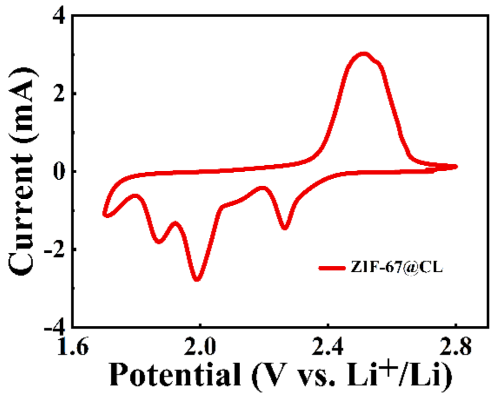

Figure S4: CV profiles at first cycle.

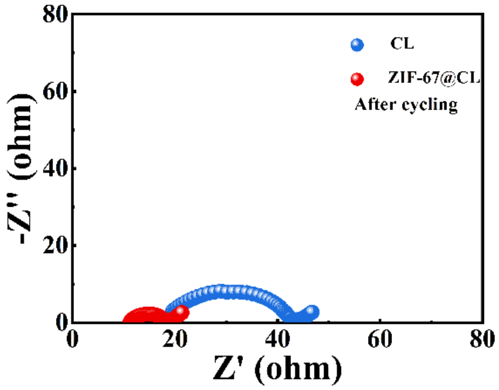

Figure S5: EIS plots after cycling.

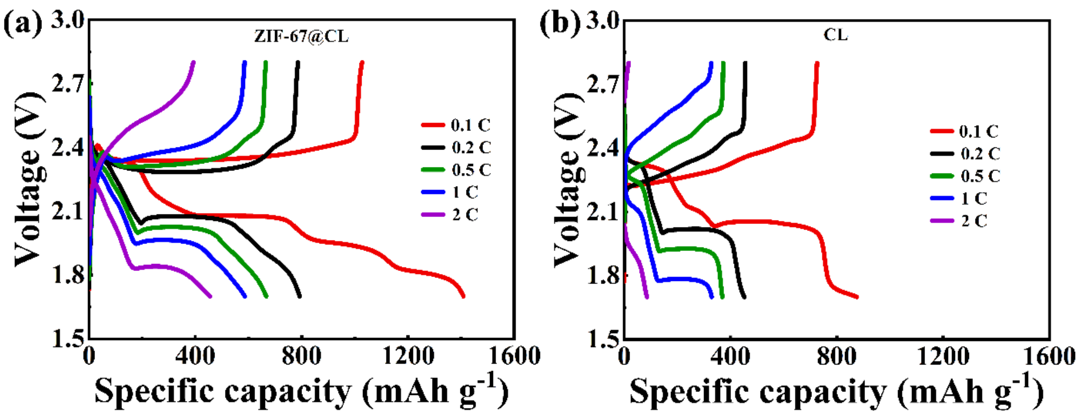

Figure S6: Rate performance correspond charge/discharge profiles of (a) S/ZIF-67@CL and (b) S/CL.

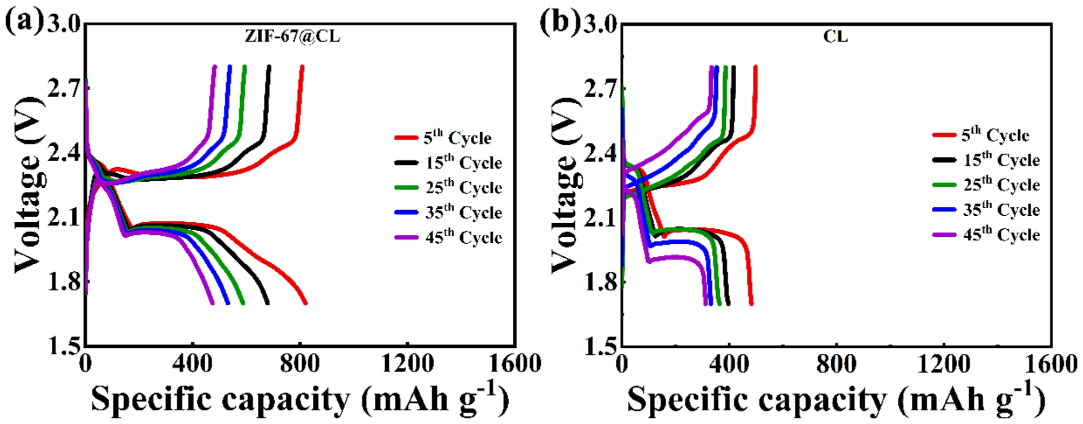

Figure S7: (a) S/ZIF-67@CL and (b) S/CL charge/discharge curves of different cycles at 0.2 C.

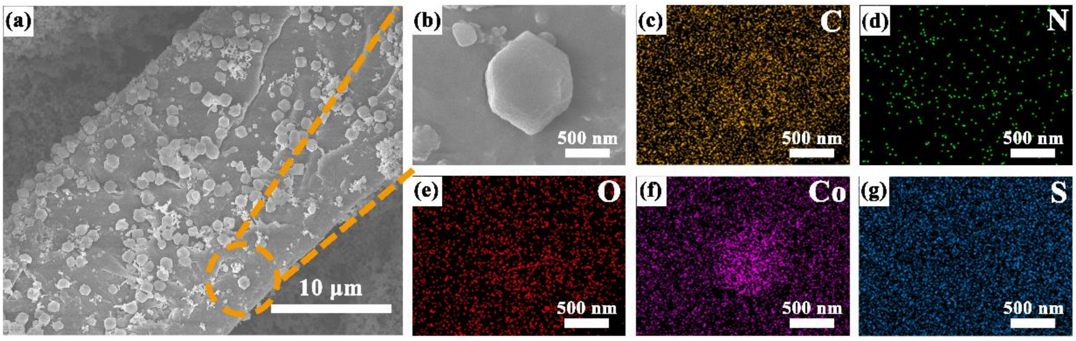

Figure S8: SEM images of (a) (b) ZIF-67@CL after Li-S batteries cycling and (c-g) corresponding elemental mappings of C, N, O, Co, S.

.

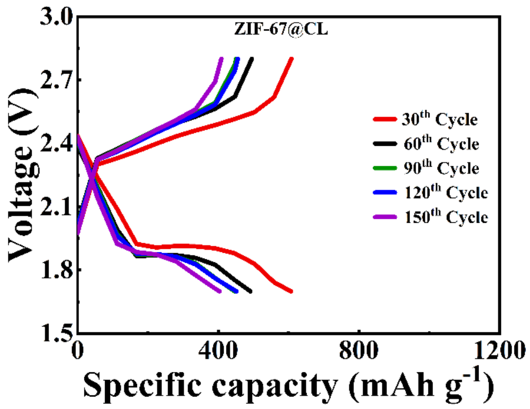

Figure S9: S/ZIF-67@CL charge/discharge curves of different cycles at 2 C.
